# A Versatile Materials Class for Solution‐Processed Optics and Photonics Based On Titanium Oxide Hydrates and Polyalcohols: A Perspective

**DOI:** 10.1002/adma.202507028

**Published:** 2025-10-23

**Authors:** Victoria Quirós‐Cordero, Alex H. Balzer, Stefan Bachevillier, Nissa Watkins, António Fernandes Ferreira, Joseph Mushyakov, Mehul Dhoot, Carlos Silva‐Acuña, Paul N. Stavrinou, Natalie Stingelin

**Affiliations:** ^1^ Department of Materials Science and Engineering Georgia Institute of Technology Atlanta Georgia 30332 USA; ^2^ Department of Chemical and Biomolecular Engineering and Center for Plastics Innovation University of Delaware Newark Delaware 19716 USA; ^3^ Department of Materials and Centre for Plastics Electronics Imperial College of London London SW7 2AZ UK; ^4^ Department of Physics Georgia Institute of Technology Atlanta Georgia 30332 USA; ^5^ School of Chemical and Biomolecular Engineering Georgia Institute of Technology Atlanta Georgia 30332 USA; ^6^ Institut Courtois & Département de Physique Université de Montréal 1375 Avenue Thérèse‐Lavoie‐Roux Montréal Québec H2V 0B3 Canada; ^7^ Department of Engineering Science University of Oxford Oxford OX1 3PD UK

**Keywords:** diffraction gratings, distributed Bragg reflectors, exciton‐polaritons, heat mirrors, high refractive index polymers, inorganic‐organic hybrid materials, metal oxide hydrates, optical microcavities

## Abstract

The ability to propagate light within a structure comprising a controlled spatial distribution of the refractive index *n* prompted the telecommunications revolution of the 20th century. More recently, progress with exploiting the flow of light has led to a broad range of light‐ and heat‐management tools, as well as novel quantum devices. This perspective discusses a new versatile class of optical materials based on molecular hybrids of metal oxide hydrates and commodity polymers, such as poly(vinyl alcohol). These fascinating, easy‐to‐produce materials are examined, and their processing into useful architectures such as photonic crystals is reviewed, with a focus on thin‐film optics. Their potential in other areas is also assessed, for instance, for the fabrication of optical microcavities that allow the formation of exciton‐polaritons, enabling studies on strong light‐matter interactions. Generally, these molecular hybrids open future opportunities in applications like optics, photonics, quantum devices, catalysis, and beyond.

## Graphical abstract

A new, solution‐processable class of optical materials based on molecular hybrids of metal oxide hydrates and commodity polymers is discussed here, including their synthesis, processing into thin films, patterning, and photonic structures realized to date. Due to their low optical loss and tunable refractive index through composition and post‐deposition treatments, this library of materials expands the capabilities to control light.

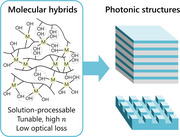

## 1 Introduction

The field of photonics has made great technological and scientific progress over the last few decades, that has built on impressive developments in passive and active (i.e., non‐emitting and emitting) photonic devices. Accordingly, photonics has become a growth area globally, which can be evidenced by the rapidly expanding optical communication, display, virtual/augmented reality, and quantum technology industries.^[^ ^1^ ^]^

Our ability to control photons (light) is, however, still in its infancy in many ways, especially when compared with how well we can manipulate electronic charge carriers. Most advancements to date have been achieved using inorganic materials, mainly metal oxides and III‐V semiconductors. The reason is that structures with very high refractive indices (high‐refractive‐index contrast),^[^ ^2^, ^3^, ^4^ ^]^ low optical loss, controlled thicknesses, and precise patterns comprising sub‐100 nm features can be readily realized. Thereby, top‐down approaches are most often employed for fabrication/patterning. For bottom‐up fabrication under ambient, bench‐top conditions, self‐assembled metallic colloids have emerged as interesting options. They also can display very high refractive indices (often n ≥ 5.0) and are of low optical loss when in the off‐resonant regime (quasi‐static regime); however, in the resonant regime, which is often in the visible spectral range, the loss can be significant.^[^ ^5^, ^6^ ^]^ Organic materials, especially polymers, have typically been limited to applications such as optical fibers, lenses, and nonlinear optics.^[^ ^7^, ^8^ ^]^ While plastics offer, generally, straight‐forward processing, frequently at high throughput, the refractive index, *n*, of organic materials is limited to at best 1.80 for a small subset of polymers with aromatic rings, sulfur‐containing groups, and halogens (except fluorine);^[^ ^9^ ^]^ most often, *n* is, however, found in a range between 1.40 and 1.65,^[^ ^2^, ^10^ ^]^ i.e., a range that does not allow the realization of a sufficiently high refractive‐index contrast in photonic structures unless anisotropic architectures, e.g., birefringent systems, are used.^[^ ^11^, ^12^ ^]^ Hence, a strong need exists for material systems that i) enable control of both the refractive index, *n*, and the refractive‐index contrast, Δ*n*, in architectures such as photonic crystals; ii) allow fabrication of devices with minimal optical loss; and, iii) can be readily fabricated and/or patterned into desired structures at low cost and/or large area.

One option for combining the desirable optical properties of inorganic materials with the benefits of polymers (e.g., simple, high‐throughput fabrication; mechanical robustness) is to use inorganic/organic systems. A common route centers around employing nanocomposites based on nanocrystalline inorganic species or nanoparticles (e.g., nanocrystalline TiO_2_, Al_2_O_3_, ZrO_2_, CeO_2_, PbS, ZnS, and ZnO) embedded in a polymer.^[^ ^13^, ^14^, ^15^, ^16^ ^]^ However, this approach can be problematic as the nanoparticles may aggregate at small loads of the inorganic component, resulting in systems with considerable haze,^[^ ^15^, ^16^ ^]^ often rendering the material fully opaque; when integrating them into photonic structures, a poor optical/photonic device performance is, consequently, obtained. Another issue is that the aggregation of the inorganic component limits the quantity that can be introduced into a polymer host and, thus, the range of *n* that can be accessed, yielding materials with a refractive index close to that of organics. Too high inorganic content can furthermore diminish processability, resulting in an additional constraint on the applicability of these materials systems in optical and photonic devices.

**Figure 1:**
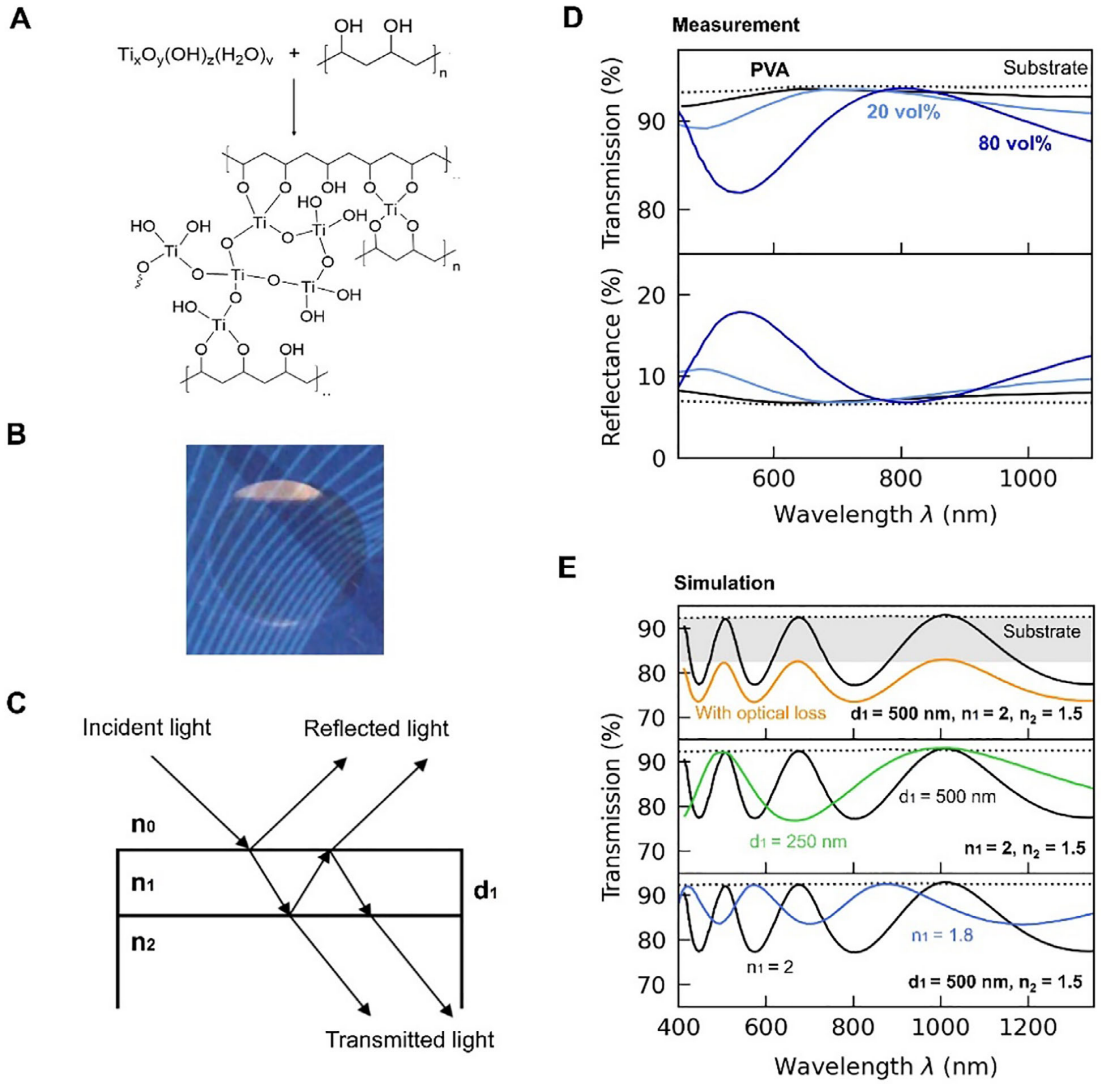
Titanium oxide hydrates/poly(vinyl alcohol) hybrid materials enable the fabrication of thin‐film structures of low optical loss. A) Condensation of titanium oxide hydrates with poly(vinyl alcohol) (PVAl) to produce molecular hybrid materials of high optical quality. B) Picture of a drop‐cast titanium oxide hydrates/PVAl hybrid film illustrating the high transparency that can be achieved, also at a high inorganic content of 50 vol%. [Figure reproduced with permission from ref. [17], Copyright 2012, Wiley‐VCH] C) In a thin film of refractive index *n*_1_ and thickness *d*_1_, incident light is transmitted and reflected at the front and back interfaces with air (*n*_0_) and the substrate (*n*_2_), respectively. D) Fabry‐Pérot oscillations measured in transmission (top) and reflection (bottom) for titanium oxide hydrates/PVAl hybrid thin films with low (20 vol%) and high (80 vol%) inorganic content illustrate the high transparency and very low optical loss of such systems. Data for neat PVAl and a bare substrate are also displayed for comparison. [Figure adapted with permission from ref. [18], Copyright 2019, Wiley‐VCH] E) Transfer matrix simulations of hypothetical scenarios to illustrate that the amplitude and period of Fabry‐Pérot oscillations contain information about the optical loss (top), thickness (middle), and refractive index (bottom) of thin films.

Molecular hybrids^[^ ^17^, ^19^, ^20^, ^21^, ^22^, ^23^, ^24^, ^25^, ^26^, ^27^ ^]^ provide promising alternatives to nanocomposites. If the inorganic species reacts with the polymer matrix, or if strong interactions form between them —e.g., via strong secondary bonds such as hydrogen bonds— aggregation of the inorganic species into particles or crystallization of either component is usually prevented. Hence, an amorphous material that is homogenous on the molecular level is obtained, with the moniker “molecular hybrid” frequently used. Due to their homogenous and amorphous nature, such hybrids are typically highly transparent. For an in‐depth review of the field of molecular hybrid materials, we refer the reader to ref. [22]. Here, we focus on inorganic/organic hybrids based on titanium oxide hydrates (“amorphous titania”, i.e., titanium compounds containing a certain number of Ti‐OH groups).^[^ ^17^, ^19^, ^21^, ^23^, ^24^, ^25^, ^26^, ^27^ ^]^ Originally described in the early 20th century, titanium oxide hydrates have attracted little consideration over recent decades—especially when compared with the well‐investigated crystalline titanium dioxide polymorphs, rutile and anatase. Yet, as we note, titanium oxide hydrates exhibit remarkable optical properties such as a relatively high refractive index (1.9 – 2.2),^[^ ^28^ ^]^ and their amorphous nature renders them fully transparent. In addition, titanium oxide hydrates undergo condensation reactions or, at minimum, form strong secondary bonds such as hydrogen bonds with “hosts” containing hydroxyl groups, leading to molecular hybrid materials.^[^ ^22^ ^]^ Some polymers that have been employed in such hybrids are poly(vinyl alcohol),^[^ ^17^ ^]^ poly(methyl methacrylate),^[^ ^21^ ^]^ acrylic resins,^[^ ^24^, ^27^ ^]^ poly(arylene ether ketone), and poly(arylene ether sulfone).^[^ ^19^ ^]^ We review the design and synthesis of molecular hybrids based on titanium oxide hydrates and poly(vinyl alcohol) (see **Figure** **1**A), the deposition methods that may be applied for single thin‐film and multilayer fabrication, their optical characterization, and their utilization to manufacture fully solution‐processed diffraction gratings, distributed Bragg reflectors (DBRs), light/heat‐management structures for solar cells, and optical cavities with exciton‐polaritons.^[^ ^17^, ^18^, ^29^, ^30^, ^31^, ^32^ ^]^ An outlook on how this material library may be extended is noted at the end of the article.

## 2 Titanium Oxide Hydrates/Poly(Vinyl Alcohol) Hybrids

### 2.1 Synthesis

The synthesis of titanium‐oxide‐hydrate‐based molecular hybrids is straightforward. It is based on a one‐pot synthesis where broadly available TiO_2_ precursors, such as titanium tetrachloride (TiCl_4_) and titanium tetraisopropoxide (TTIP), are first dissolved in H_2_O to undergo hydrolysis, yielding a solution comprising inorganic species of predominantly mono‐nuclear nature (e.g., hydroxides or chlorides), provided the temperature is maintained at 0 °C.^[^ ^29^ ^]^ These hydrolysis solutions can then be added to aqueous solutions of polymers with a large number of hydroxyl groups, such as poly(vinyl alcohol). The polymer “host”/matrix stabilizes the inorganic species, preventing the formation of (crystalline) nanoparticles and resulting in titanium oxide hydrates (see Figure 1A). These are stable if the solutions are continued to be kept at low temperatures.^[^ ^17^ ^]^ If needed, acids can be added to lower the solutions’ pH, which further stabilizes the inorganic species.^[^ ^17^ ^]^ As a result, the solutions are stable for months when stored in a fridge and readily available for filmmaking and casting as needed. Thereby, the viscosity of the solutions can be tuned via the selection of the polymer content in the solution and the polymer's molecular mass in order to comply to the coating/deposition methods’ needs.

Desirably, the inorganic content in the final (solid) titanium oxide hydrates/PVAl hybrids can be readily adjusted to control properties. This is done by either mixing the hydrolysis and the polymer solutions in different proportions while keeping the polymer and inorganic content of each solution constant, or by varying the concentration of either solution but keeping the same mixing ratio (in volume) of the hydrolysis solution and PVAl solution. The *nominal* volume fraction of titanium oxide hydrates, *V*_HyTi_, in the final titanium oxide hydrates/PVAl hybrids can then be estimated as:^[^ ^17^, ^18^ ^]^

(1) $$\;V_{\mathrm{HyTi}}=100\cdot \frac{\left(\frac{M_{\mathrm{HyTi}}}{\rho_{\mathrm{HyTi}}}\right)\cdot c_{\mathrm{HyTi}}}{\left(\frac{M_{\mathrm{HyTi}}}{\rho_{\mathrm{HyTi}}}\right)\cdot c_{\mathrm{HyTi}}+\left(\frac{1}{\rho_{\mathrm{PVAl}}}\right)}$$

using *M*_HyTi_ = 90 g/mol and *ρ*_HyTi_ = 1.95 g/cm^3^ for the molar mass and density of titanium oxide hydrates (“hydrated titania”), respectively;^[^ ^33^ ^]^ *ρ*_PVAl_ is the density of poly(vinyl alcohol), and *c*_HyTi_ is the titanium oxide hydrates content expressed in mol Ti/g PVAl. **Table** **1** summarizes the volume fractions corresponding to a certain value of mol Ti/g PVAl. [Note: Titanium oxide hydrates/PVAl hybrids with up to 90 vol% titanium oxide hydrates can be synthesized without visible nanoparticle formation.]

**Table 1:** Compositions of titanium oxide hydrates/poly(vinyl alcohol) hybrid materials, calculated from mmol Ti/g PVAl to a *nominal* volume fraction of titanium oxide hydrates.

[mmol Ti/g PVAl]	*V*_HyTi_ [vol %]
0.9	5
4.3	20
11.5	40
25.8	60
68.8	80

### 2.2 Thin‐Film Fabrication

A major requirement for the creation of photonic structures is ease of fabrication. Titanium oxide hydrates/PVAl solutions can be readily cast into films. This is demonstrated by the example of a drop‐cast film made from a system with 50 vol% inorganic content, shown in Figure 1B. Since the titanium oxide hydrates and the PVAl undergo condensation reactions during film drying, leading to crosslinks between the organic and inorganic species, the resulting films are amorphous limiting light scattering. The films also become insoluble in water and organic solvents even at low inorganic content (<1 vol%).^[^ ^17^ ^]^ Favorably, the condensation reaction can be promoted by thermal annealing procedures, providing a “knob” to fine‐tune the refractive index, as discussed in section 2.3.

**Figure 2:**
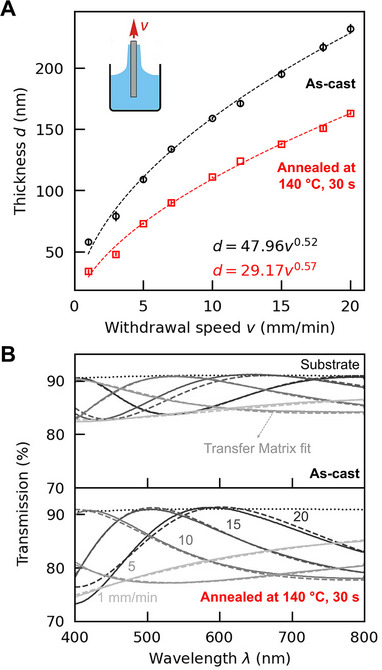
Reproducible thicknesses with sub‐10 nm resolution are achieved when dip‐coating titanium oxide hydrates/poly(vinyl alcohol) hybrid thin films. A) Calibration curves illustrate the average thickness as a function of withdrawal speed, *v*, measured for dip‐coated films of a titanium oxide hydrates/PVAl hybrid (60 vol% inorganic content), cast from a solution prepared by mixing an aqueous 1 M hydrolysis solution with a 3 wt.% aqueous solution of PVAl (weight‐average molecular weight, *M*_w_ ≈130 kg mol^−1^; residual content of acetyl groups = 10%–12%). Data is presented for as‐cast (black circles) and annealed (red squares) films. The error bars indicate standard deviations below 4 nm, corresponding to four films fabricated at each speed and measured at five different locations per sample (the center and four corners), demonstrating both excellent thickness reproducibility among multiple films and their homogeneity. Power laws (dashed lines) are fitted to the average values to interpolate the films’ thicknesses at other withdrawal speeds. B) Optical transmission of these dip‐coated films: as‐cast (top) and annealed at 140 °C for 30 s on a high‐precision hotplate after drying (bottom). The thickness and refractive index of the films are determined by fitting a transfer matrix model (dashed lines) to the observed Fabry‐Pérot oscillations (solid lines). [Figure reproduced with permission from ref. [34], Copyright 2024, Victoria Quirós‐Cordero.].

As importantly, varying the solution viscosity and specific processing conditions—for example, the withdrawal speed, *v*, during dip‐coating (see inset in **Figure** **2**A)—a desired film thickness, *d*, can be produced in a highly exact fashion.^[^ ^35^, ^36^ ^]^ This requires the establishment of calibration curves of “thickness as a function of withdrawal speed” (at constant viscosity), as well as curves of “thickness as a function of viscosity” (at constant *v*). Two examples of such calibration curves are presented in Figure 2A, for as‐cast films and films annealed post‐deposition at 140 °C for 30 s, both prepared with a hybrid containing 60 vol% titanium oxide hydrates. Using the data from these calibration curves, the thickness, *d*, of titanium oxide hydrates/PVAl hybrid films can be controlled with sub‐10 nm precision in a reproducible manner, with standard deviations of less than 4 nm at all withdrawal speeds.^[^ ^18^, ^32^ ^]^ This excellent thickness control permits interpolation to other withdrawal speeds by fitting the average film thickness values to a power law that minimizes the least squares (dashed lines in Figure 2A). Other information can be deduced. For instance, from the data shown in Figure 2A, it can be concluded that thermal annealing, which promotes further condensation of the titanium oxide hydrates with PVAl, densifies the material^[^ ^17^, ^18^, ^29^, ^30^, ^31^, ^32^, ^37^ ^]^ and results in thinner films.

Another key attribute of titanium oxide hydrates/PVAl hybrid films is that they display excellent optical quality. This is evident from simple optical transmission measurements, where constructive and destructive interference between transmitted light at the films’ interfaces (Figure 1C) result in Fabry‐Pérot oscillations. These oscillations are clearly seen in the transmission spectra obtained for films composed of hybrids with varying inorganic content (Figure 1D) or produced with the same hybrid composition (60 vol% titanium oxide hydrates) but at different withdrawal speeds (Figure 2B). The high transparency (low optical losses) of all films can be inferred from the fact that the transmission maxima of the Fabry‐Pérot oscillations align with the transmission of the bare substrate (dotted lines in Figure 1D and Figure 2B). According to transfer matrix model (TMM) simulations of hypothetical scenarios (high versus low optical loss; high‐ versus low‐refractive‐index films; thick versus thin layers; etc.), this occurs only for materials with negligible optical loss (Figure 1E, top panel), which aligns with the observation of no absorption and scattering, as experimentally deduced from the relationship between the recorded transmission (T) and reflection (R), which satisfies: T + R = 1 (Figure 1D). [Note: Use of withdrawal speeds of 1 and 5 mm/min results in very thin films, rendering Fabry‐Pérot oscillations with full periods that are too long to be resolved within the analyzed wavelength range (Figure 1E, middle panel). However, TMM simulations, based on a model with no loss (dashed lines in Figure 2B), correlate very well with the experimentally measured transmission, from which the conclusion can be drawn that these thin films are also of excellent optical quality.]

### 2.3 Tunable Refractive Index

**Figure 3:**
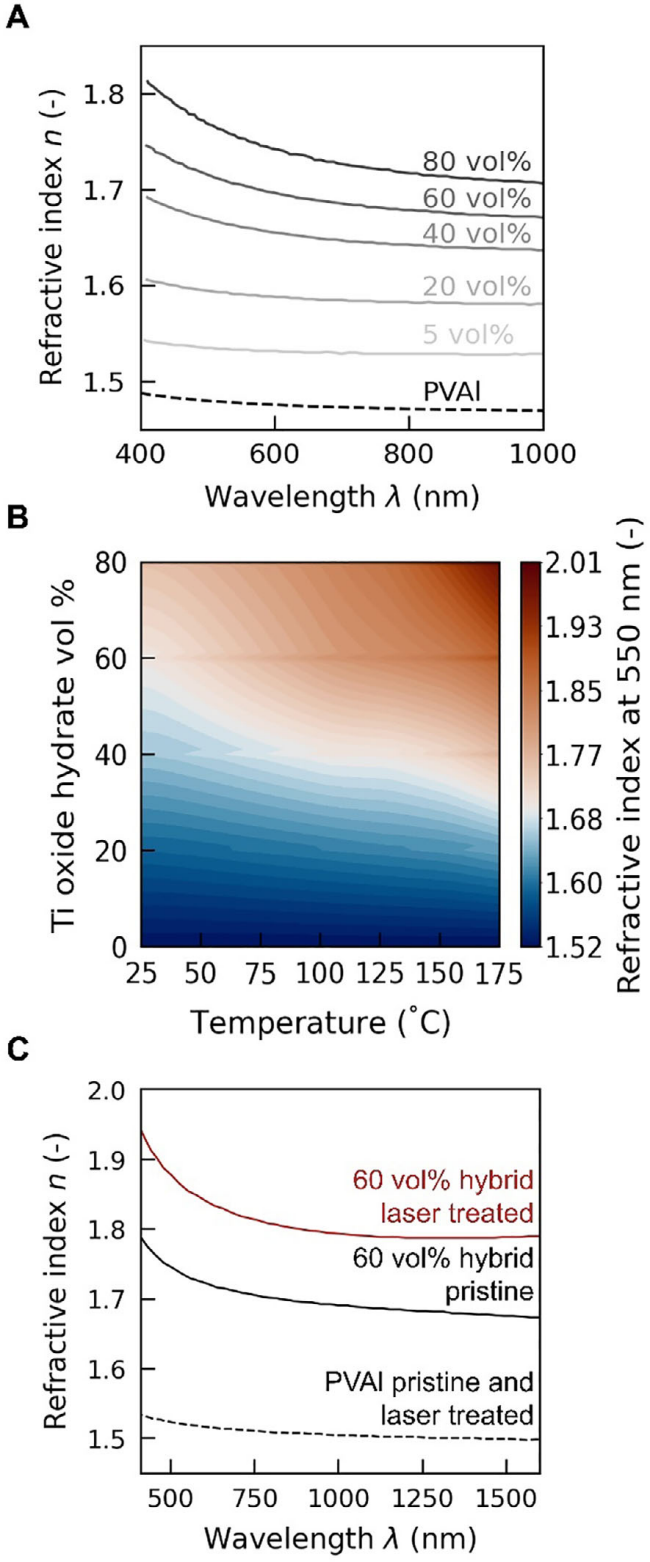
Tunability of the refractive index of titanium oxide hydrates/PVAl hybrids based on composition, thermal annealing, and laser patterning. A) Refractive index of as‐cast thin films with varying inorganic content. B) 2D map illustrating the tunability of the hybrid's refractive index at 550 nm with respect to both composition and annealing temperature. Refractive indices indicated in red tones correspond to values exceeding 1.70, a value typically unattainable with organic materials. [Figure reproduced with permission from the Supporting Information of ref. [32], Copyright 2023, Wiley‐VCH.] C) Increase in refractive index of titanium oxide hydrates/PVAl hybrid films (60 vol% inorganic content) when subjected to an ultrafast 515 nm laser. The refractive index of PVAl remains unchanged under the same exposure conditions. [Figure reproduced under the terms of CC‐BY license from ref. [31], Copyright 2022, The Authors, published by Wiley‐VCH].

As in many inorganic/organic systems, including nanocomposites,^[^ ^13^, ^14^, ^15^, ^16^ ^]^ the refractive index of titanium oxide hydrates/PVAl hybrid materials is determined by the inorganic content. A qualitative insight into this trend can be easily obtained from the Fabry‐Pérot oscillations, discussed in Section 2.2 and measured on different titanium oxide hydrates/PVAl hybrid films. While an increase in film thickness (at a constant refractive index) leads to oscillations of higher frequency (see the TMM simulations presented in Figure 1E, middle panel), a higher refractive index (at a constant thickness) produces oscillations with larger amplitude and shorter period (Figure 1E, bottom panel). This can be experimentally observed when comparing the transmission of 220 nm‐thick neat PVAl films with that of titanium oxide hydrates/PVAl hybrids with inorganic contents of 20 and 80 vol% and the same thickness (Figure 1D). More quantitatively, the refractive index and film thickness are determined by fitting a transfer matrix model (dashed lines, Figure 2B) to the Fabry‐Pérot oscillations measured in thin films in transmission (solid lines, Figure 2B).^[^ ^17^, ^18^, ^29^, ^30^, ^31^, ^32^, ^37^ ^]^ Thereby, it is assumed that the refractive index follows the empirical Cauchy equation.^[^ ^38^ ^]^  It is found that the refractive index of *as‐cast* titanium oxide hydrates/PVAl films can be tuned between 1.47 and 1.76 with composition, in good agreement with ellipsometry data (values taken at 550 nm; see Figure 3A,B). Moreover, only a relatively weak refractive‐index dispersion with wavelength is observed (Figure 3A,C). [Note: More elaborate refractive‐index modeling for homogeneous systems is challenging to apply to the molecular hybrids, which are polar, amorphous, crosslinked networks where each species of atoms can exhibit different bonding and interactions, and consequently, atomic polarizabilities.^[^ ^39^ ^]^ Similarly, models developed for composite materials, with distinct high‐ and low‐refractive‐index phases,^[^ ^40^, ^41^ ^]^ cannot be applied due to the highly homogenous, amorphous nature of the titanium oxide hydrates/PVAl hybrids.]

A rather unique means for the design and fabrication of optical devices and photonic structures is made available because annealing leads to an increase of refractive index. Accordingly, the Fabry‐Pérot oscillations of annealed films exhibit a larger amplitude than those of their as‐cast counterparts produced with the same solutions and under the same conditions. However, their period is longer than that of the oscillations from as‐cast films. These experimental findings imply that thermal annealing increases the material's refractive index and reduces the film's thickness. Both effects are attributed to the densification of the films, as already mentioned above.^[^ ^17^, ^18^, ^29^, ^30^, ^31^, ^32^, ^37^ ^]^

**Figure 4:**
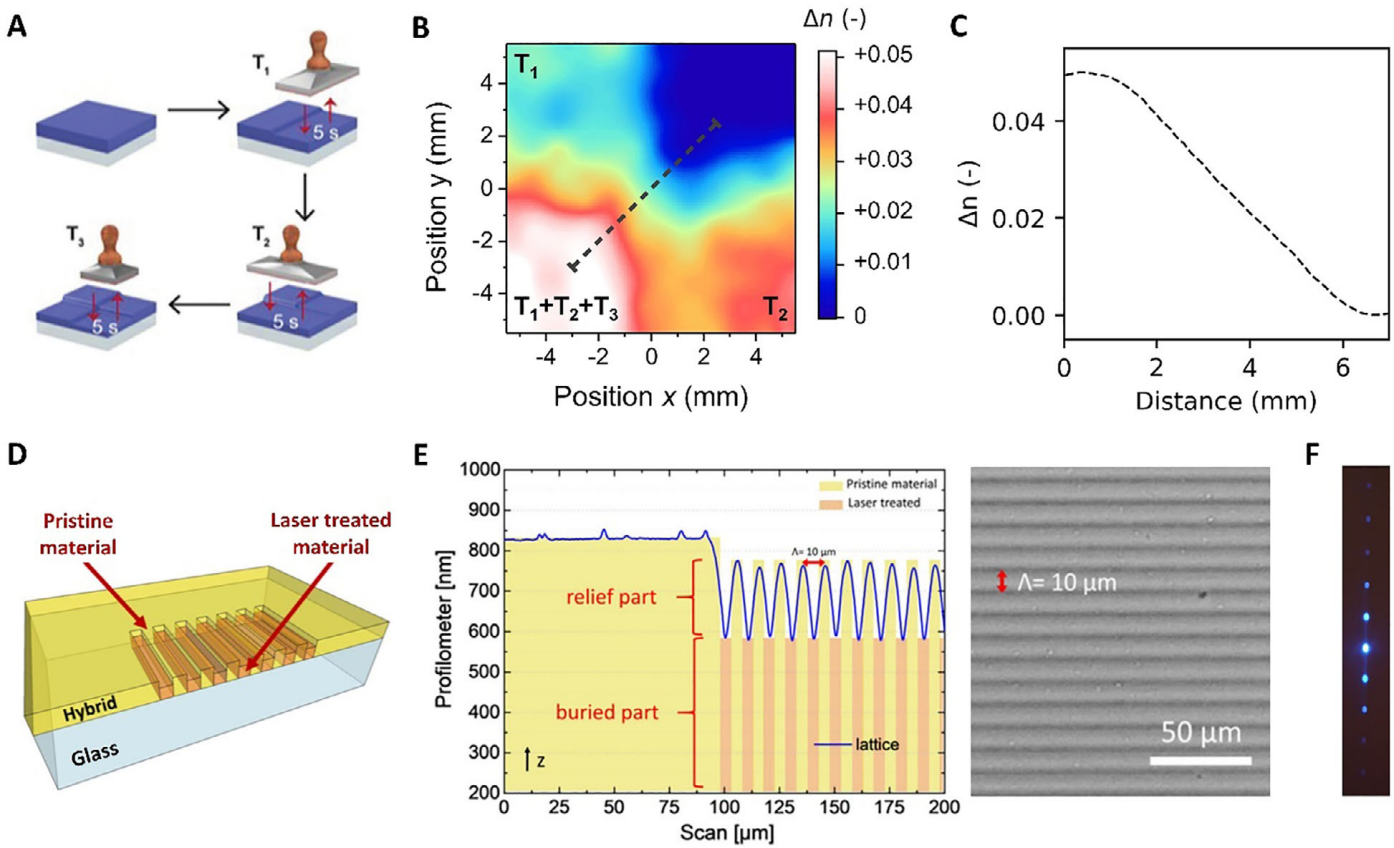
Patterning 2D structures in titanium oxide hydrates/poly(vinyl alcohol) hybrid films via hot‐stamping and femtosecond laser writing. A) Hot‐stamping sequence followed to create four features with distinct refractive indices in a titanium oxide hydrates/PVAl hybrid film (60 vol% inorganic content). The flat metallic stamp was preheated to different temperatures (*T*_1_ = 50 °C, *T*_2_ = 100 °C, *T*_3_ = 150 °C) and brought into contact for 5 s with different film sections. B) Resulting pattern with four regions of different refractive index. C) Spatial profile of the refractive‐index change between the two features of the highest refractive‐index contrast. D) Fabrication of an optical diffraction grating in a titanium oxide hydrates/PVAl hybrid film (60 vol% inorganic content) via femtosecond laser writing. E) Profilometer data (left) and optical micrograph (right) of the pattern realized. F) Symmetrical multi‐order diffraction pattern produced in transmission by the optical grating. [Panels A–C were reproduced with permission from ref. [30], Copyright 2022, The Royal Society of Chemistry; and panels D‐F were adapted under the terms of CC‐BY license from ref. [31], Copyright 2022, The Authors, published by Wiley‐VCH].

The maximum refractive index achieved to date is 2.01 (at 550 nm; Figure 3B), which is a significantly higher value than what can be obtained with purely polymer‐based materials.^[^ ^2^, ^9^, ^10^ ^]^ Advantageously, a high refractive index is achieved without introducing optical loss, such as absorption or scattering. Moreover, *n* can be fine‐tuned via the selection of the annealing temperature.^[^ ^15^, ^16^ ^]^ This beneficial feature enables, e.g., the creation of refractive‐index patterns.^[^ ^18^, ^30^ ^]^ Indeed, graded refractive‐index structures were produced, exploiting that the heat‐induced refractive‐index changes are additive, as illustrated in **Figure** **4**A–C. Specifically, a refractive‐index pattern containing four regions of distinct *n* was reported, fabricated by consecutively heating specific areas of a film, cast from a titanium oxide hydrates/PVAl hybrid of 60 vol% inorganic content, to different temperatures (Figure 4A,B). In order to achieve rapid treatment to limit lateral heat flow, microcontact photothermal annealing^[^ ^30^ ^]^ or similar procedures^[^ ^42^, ^43^ ^]^ might be applied, which so far led to refractive‐index changes, Δ*n*, of up to +0.06 with a spatial resolution of ≈50 µm.^[^ ^30^ ^]^

Another method to adjust the refractive index of titanium oxide hydrates/PVAl hybrid films is through exposure to a laser beam — that is, laser writing or patterning.^[^ ^31^, ^44^, ^45^ ^]^ For example, using a 515 nm fs‐laser, the refractive index of titanium oxide hydrates/PVAl hybrid films can be locally increased upon irradiation, as depicted in Figure 4D. Concurrently, a reduction in thickness is observed in the exposed areas. This process, tentatively attributed to local heating via two‐photon absorption, facilitates the creation of a 10 µm‐pitch phase grating within a 5 × 5 mm^2^ area (Figure 4E) that, when functioning in transmission mode, produces symmetrical multi‐order diffraction in relation to the incident beam's zero order (Figure 4F).^[^ ^31^ ^]^ [Note: Titanium oxide hydrates/PVAl hybrid films, both as‐cast and post‐processed via partial annealing and laser patterning, have thicknesses and optical properties (e.g., refractive index values and negligible loss) that are stable in *ambient* conditions. Exposure to 150 °C for a few seconds fully anneals the titanium oxide hydrates/PVAl hybrids; i.e., no further thickness changes or evolution of other material properties are observed upon continued exposure to heat.^[^ ^18^, ^30^ ^]^ Therefore, highly stable structures can be produced with fully annealed hybrids.]

## 3 Fully Solution‐Processed Photonic Crystals and Optical Structures

Photonic crystals are intriguing objects due to their capacity to control electromagnetic radiation (e.g., its wavelength, amplitude, phase, propagation, and polarization), as noted by Lord Rayleigh in 1887.^[^ ^11^, ^12^, ^46^, ^47^, ^48^, ^49^, ^50^, ^51^ ^]^ Photonic crystals are composed of periodic structures that modulate the refractive index in one or more directions. The combination of a sufficiently high refractive index modulation, Δ*n*, and periodicities that induce interference of electromagnetic radiation enables the manipulation of light and the fabrication of a multitude of devices, including 1D photonic crystals, such as distributed Bragg reflectors, and 2D structures that can produce diffraction and waveguiding functionalities, among others.

### 3.1 Distributed Bragg Reflectors (DBRs)

**Figure 5:**
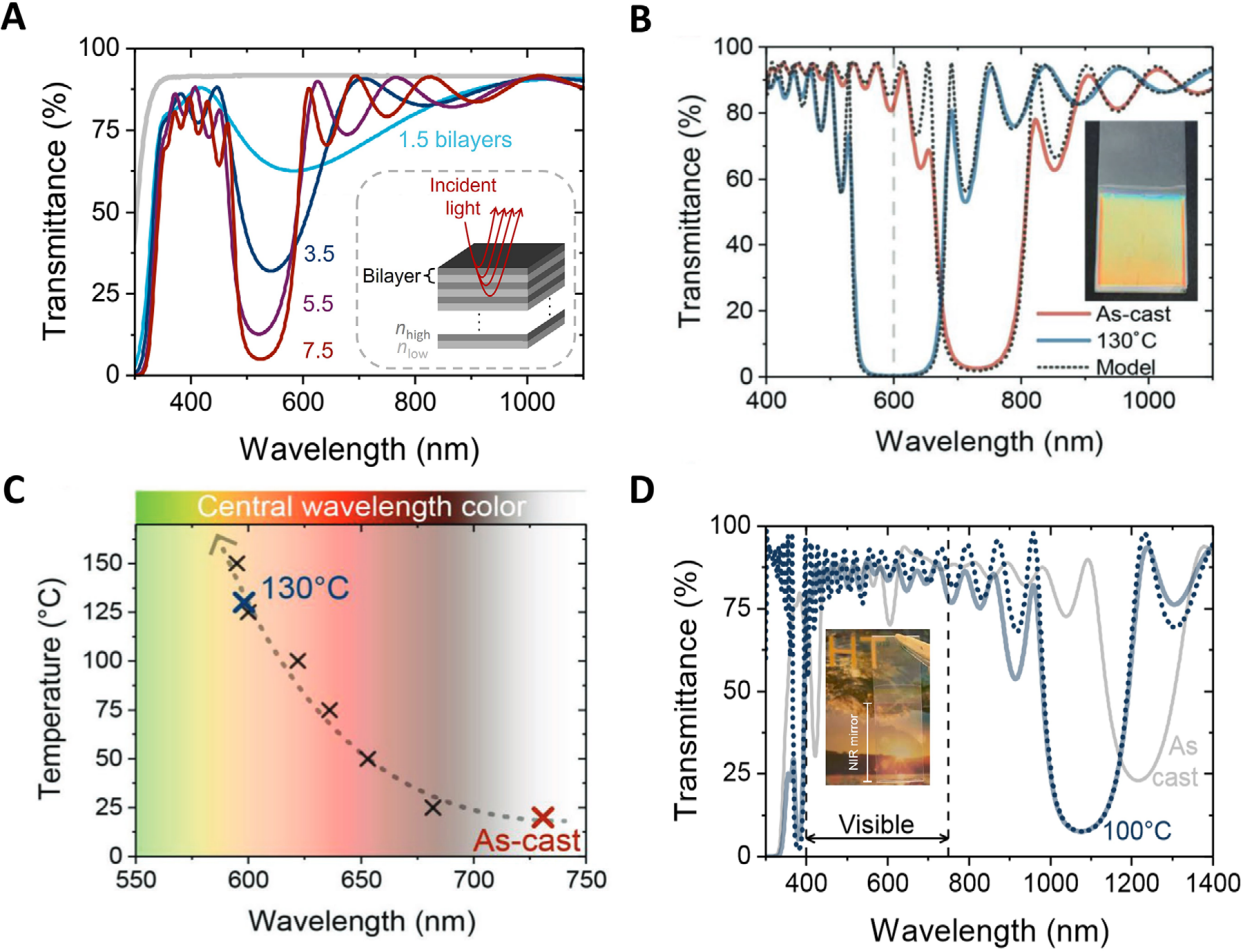
Distributed Bragg reflectors (DBRs) for the visible and NIR wavelength regimes. A) Transmittance spectra of dip‐coated quarter‐wavelength DBRs with varying numbers of bilayers, comprising high‐ (*n*_high_) and low‐refractive‐index (*n*_low_) layers, produced from a titanium oxide hydrates/PVAl hybrid (80 vol% inorganic content) and poly(methyl methacrylate), PMMA, respectively. The schematic in the inset illustrates how, at each interface, light of wavelengths centered around *λ*_B_ (red rays) is reflected. Constructive interference results in the overall reflectance of the stack. An increase in the number of DBR bilayers leads to higher reflectance. B) The reflectance and stopband width can be enhanced post‐fabrication through treatments that raise the hybrid's refractive index, such as thermal annealing. These treatments also cause the hybrid layers to contract, inducing a blueshift of the stopband. This is demonstrated for a 10.5‐bilayer DBR, consisting of a titanium oxide hydrates/PVAl hybrid with 60 vol% inorganic content (*n*_high_) and poly[4,5‐difluoro‐2,2‐bis(trifluoromethyl)‐1,3‐dioxole‐co‐tetrafluoroethylene], PFP (*n*_low_), with the entire DBR stack annealed at 130 °C. Transfer matrix model simulations (dotted lines), based on the thickness and refractive index of individual hybrid films treated under the same conditions as the DBR, accurately predict the increase in reflectance and the broadening and blueshift of the stopband. C) The blueshift of the DBR stopband upon annealing at temperatures ranging from 25 °C to 150 °C can be accurately predicted via TMM simulations (dotted line) and, thus, can be utilized for thermal sensing. D) DBRs with stopbands in the near‐IR and high transparency in the visible wavelength regime can be produced with titanium oxide hydrates/PVAl hybrids if thicker DBR layers are employed. Such structures can be used in passive cooling applications. [Panels B and C were reproduced with permission from ref. [18], Copyright 2019, Wiley‐VCH].

One of the most conceptually straightforward examples of photonic crystals involves a multilayer stack of alternating high‐ and low‐refractive‐index materials (*cf*. inset in **Figure** **5**A).^[^ ^11^, ^47^, ^52^, ^53^ ^]^ Often referred to as “1D crystals”, these dielectric mirrors (also known as distributed Bragg reflectors) are commonly used in lasers and telecommunication technologies.^[^ ^52^, ^53^ ^]^ The reason is that they provide high reflectivity over a selected, well‐defined range of wavelengths, which can be precisely controlled by the stack characteristics. This stands in stark contrast to metallic mirrors, which are typically not sufficiently selective regarding the wavelengths they reflect and tend to exhibit non‐zero absorption in the visible and near‐infrared regimes, impairing their reflectivity.

The key design variables for DBRs are i) the refractive index contrast (Δ*n*_i_ = *n*_i,high_ – *n*_i,low_) between, and ii) the thicknesses, *d*_i,high_ and *d*_i,low_ of, the high‐ and low‐refractive‐index layers. These parameters dictate the optical path (*n*_i_ ⋅ *d*_i_) traversed, and the phase of the reflected and transmitted components of the electromagnetic field at each interface.^[^ ^54^, ^55^ ^]^ Partial interfacial reflections that combine constructively explicitly build up the structure's reflectance, as shown in the inset of Figure 5A. The greater the refractive index contrast between adjacent layers, the more light is reflected per interface. Similarly, increasing the number of high‐ and low‐refractive‐index bilayers enhances the final structure's reflectance. This is illustrated by the example of quarter‐wavelength DBRs produced with a titanium oxide hydrates/PVAl hybrid (80 vol% inorganic content) and poly(methyl methacrylate), PMMA, functioning as the high‐ and low‐refractive‐index materials, respectively (Figure 5A). DBRs comprising 3.5 bilayers transmit less than 35% of the incoming light (reflect more than 65%), while structures with 7.5 bilayers reflect over 95%. [Note: In quarter‐wavelength DBRs, the reflected central wavelength, or Bragg wavelength, *λ*_B_, satisfies the relation: *n*_i_(*λ*_B_)⋅*d*_i_ = *λ*_B_ */*4].

Beneficially, the changes in refractive index and thickness observed for the titanium oxide hydrates/PVAl hybrid upon thermal annealing remain essentially identical for a single‐layer film or individual layers embedded in a DBR stack. In addition, the short annealing times of ≤30 s necessary to increase the refractive index of the titanium oxide hydrates/PVAl hybrids *do not* affect the low‐refractive‐index material (neither *n*_low_ nor *d*_low_), in particular when solution‐processable fluorinated polymers such as poly[4,5‐difluoro‐2,2‐bis(trifluoromethyl)‐1,3‐dioxole‐co‐tetrafluoroethylene], PFP, or the commercial Cytop™ are used. Hence, the entire DBR structures can be heat‐treated to induce a higher refractive‐index contrast between the high‐ and low‐refractive‐index layers realized via an increase of *n*_high_.^[^ ^17^, ^18^, ^32^ ^]^ Accordingly, a targeted reflectivity can be achieved in thermally annealed DBRs using fewer layers in the stack compared to untreated DBRs. The higher *n*_high_ and, hence, the higher Δ*n* = *n*_high_ – *n*_low_, also leads to a broadening of the stopband, as observed in the transmission spectra of the 10.5‐bilayer DBR, produced with PFP and a titanium oxide hydrates/PVAl hybrid with 60 vol% inorganic content, presented in Figure 5B. An excellent reflectivity of ≈97% can already be realized in the as‐cast DBR, with a stopband full width at half maximum, ∆*λ*, of ≈138 nm (red spectrum). After annealing the entire DBR stack at 130 °C for a brief period, the reflectivity of the DBR increases to above 99% (blue spectrum). Moreover, the full width at half maximum of the stopband increases to ≈149 nm. A blue shift of the stopband was also reported and attributed to the reduction in the thickness of the high‐refractive‐index layers upon annealing.

It is noteworthy that the transfer matrix model accurately describes the optical response of these stacks when using the refractive index and thickness of individual films processed and treated under the same conditions as the entire DBR (Figure 5B, dashed lines),^[^ ^17^, ^18^, ^32^ ^]^ demonstrating the exceptional reproducibility of layer thickness and refractive index within the DBR structures. This precision in simulating the characteristics of the DBR, including after annealing, can be leveraged for all‐optical temperature sensing (Figure 5C). Depending on the temperature at which the DBR is heat‐treated, it will exhibit a unique reflection intensity and stopband position that can be rather precisely predicted.

Principally, many applications can benefit from the availability of easy‐to‐coat DBRs, including filters, splitters, and reflectors, as well as light‐ and heat‐management structures for a range of optoelectronic devices (*cf*. refs. [52, 53]). One interesting example of this is the use of such structures in semitransparent solar cells for greenhouse integration. These solar cells exhibit relatively high transmission at wavelengths where chlorophyll, the primary pigment in plants, absorbs. Large‐area DBRs may, thus, be employed to reflect light back into the solar cell for more photons to reach the active layer, thereby increasing device efficiency. At the same time, plant growth is unaffected because the DBRs are designed not to hamper the solar cell's high transmission in the chlorophyll absorption regime.^[^ ^37^ ^]^ Another option is to produce DBRs that reflect in the NIR to IR to create heat mirrors. The intriguing aspect here is that multilayer stacks can be fabricated that remain fully transparent in the visible (see transmission spectra presented in Figure 5D) because both the titanium oxide hydrates/PVAl hybrid material and the low‐refractive‐index polymer components, such as PMMA and PFP, are non‐absorbing in a broad wavelength range above ≈350 nm. The high transparency can be seen by eye from the picture of such a titanium oxide hydrates/PVAl hybrid:PMMA DBR coated onto a glass slide provided in the inset of Figure 5D. The reflection in the NIR is ≈90%, despite the DBR consists of only 7.5 bilayers.

### 3.2 Planar Optical Microcavities

**Figure 6:**
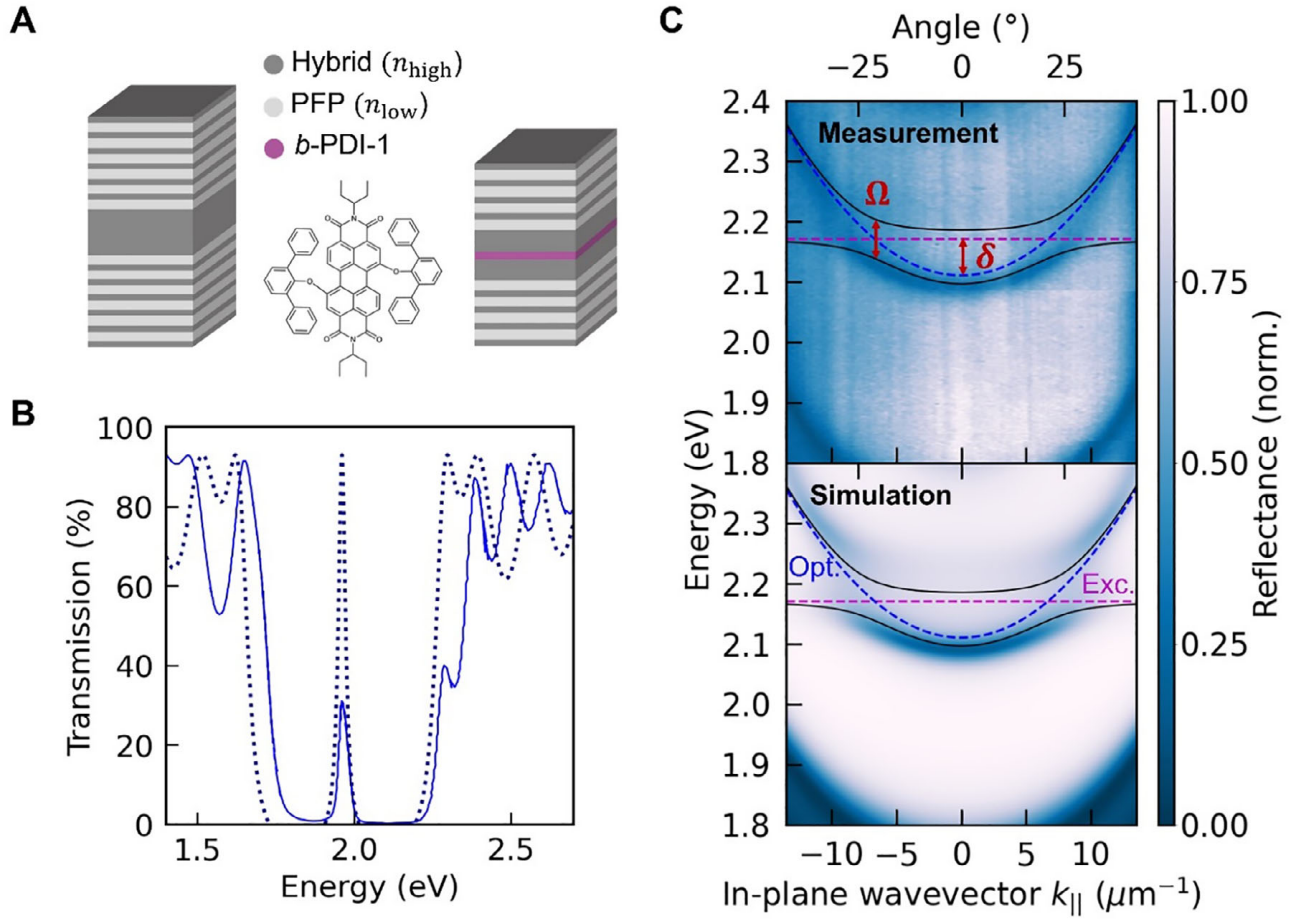
Fully solution‐processed planar optical microcavities. A) Passive and active microcavities (left and right) produced with two 6‐bilayer and two 4‐bilayer DBRs, respectively. Both cavities were fabricated with a titanium oxide hydrates/PVAl hybrid (60 vol% inorganic content; annealed at 140 °C) and PFP as the high‐ and low‐refractive‐index layers. The DBRs of the passive microcavity were separated by a titanium oxide hydrates/PVAl hybrid spacer, also with a 60 vol% inorganic content and annealed at 140 °C. The DBRs of the active microcavity used two spacers made of a titanium oxide hydrates/PVAl hybrid (60 vol% inorganic content; annealed at 140 °C) and a 30‐nm layer of a perylene‐diimide derivative, *b*‐PDI‐1. B) Transmission of the microcavity displaying an optical mode with *E*_Opt._ = 1.962 eV (*λ*_Opt._ ≈ 632 nm), represented by a solid blue line, which aligns well with its design via the transfer matrix model (TMM), shown as a dark blue dotted line. C) The energy dispersion of the active microcavity, measured in reflectance (top), reveals two exciton‐polariton states (blue halos). This measurement correlates closely with the energy dispersion in reflectance simulated by the TMM (bottom). The observed polariton states coincide with those of a Jaynes‐Cummings Hamiltonian, exhibiting a Rabi splitting *Ω* = 2⋅*g* = 65 meV (solid black lines), where *g* represents the light‐matter coupling constant, supporting strong light‐matter coupling. [Figure adapted with permission from ref. [32], Copyright 2023, Wiley‐VCH].

Optical cavities are resonant structures that confine electromagnetic radiation spatially.^[^ ^56^, ^57^ ^]^ A planar Fabry‐Pérot cavity achieves this with two parallel, high‐reflectivity surfaces separated by a spacer layer, where a standing wave forms due to interference between electromagnetic waves traveling in opposite directions.^[^ ^56^, ^57^ ^]^ A planar microcavity, so named as the spacer (or cavity) region is often just a few optical wavelengths thick (see schematic in **Figure** **6**A, left), operates on the principle that the frequency of the standing wave is determined by the optical length of the spacer. At this frequency, known as the “cavity mode” or “optical mode”, the structure becomes largely transmissive, as the example of the solution‐processed microcavity produced with titanium oxide hydrates/PVAl hybrids and PFP in Figure 6B shows (the optical mode is at ≈1.9 eV). Due to the reduced dimensions of the microcavity, it is possible to place an emissive material directly at an antinode of the electric field (Figure 6A, right), consequently enhancing the coupling of light and matter states.^[^ ^56^, ^57^ ^]^

Over the last few decades, the underlying physics of microcavity structures has been the subject of intense interest, leading to devices such as resonant‐cavity light‐emitting diodes (RCLEDs)^[^ ^58^, ^59^, ^60^, ^61^ ^]^ and vertical‐cavity surface‐emitting lasers (VCSELs);^[^ ^62^, ^63^, ^64^ ^]^ intriguing results concerning the entanglement of matter and radiation in the quantum regime^[^ ^65^, ^66^ ^]^ have also been reported. Traditionally, the mirrors employed in optical microcavities are either metallic films or inorganic DBRs fabricated via thermal deposition or sputtering,^[^ ^57^ ^]^ though a number of examples exist where solution‐processed DBRs were utilized.^[^ ^67^, ^68^, ^69^, ^70^, ^71^, ^72^, ^73^, ^74^ ^]^

Through microcavity design, optical modes at specific wavelengths, *λ*_Opt._, or energies, *E*_Opt._, can be targeted. The reason is that planar microcavities satisfy the following resonance condition:

(2) $$\sum_{i}n_{\mathrm{cavity},i}\left(\lambda_{\mathrm{Opt}.}\right)\;d_{\mathrm{cavity},i}=\left(\frac{m\lambda_{\mathrm{Opt}.}}{2}\right)$$

where m is an integer number, *n*_cavity,i_ is the refractive index of the *i*‐th material or medium between the mirrors and *d*_cavity,i_ is the distance between the mirrors occupied by the *i*‐th material or medium. Note also that the confinement of the electromagnetic field along the axis perpendicular to the mirrors (i.e., out‐of‐plane) and momentum conservation causes the optical modes to have an energy dispersion as a function of output angle or in‐plane wavevector ($\vec{k_{∥}}$):

(3) $$E_{\mathrm{Opt}.}\;\;\left(\vec{k_{∥}}\right)\;\;\approx \;\;E_{\mathrm{Opt}.}\;\;\left(\vec{k_{∥}}=\vec{0}\right)\;\;+\;\;\frac{\hbar^{2}{\left|\vec{k_{∥}}\right|}^{2}}{2m_{\mathrm{Opt}.}}$$

where *m*_Opt._ is the effective mass of the confined optical mode photons; drawing on concepts familiar from electronic band theory. In addition, confined optical modes ultimately leak and suffer from damping. The quality factor of a microcavity serves as an inverse metric of these losses (i.e., damping of the standing wave) and is given by *Q = E*_Opt._ $\left(\vec{k_{∥}}=\vec{0}\right)$/Δ_FWHM_(*E*_Opt._); this means that the smaller the optical losses, the higher *Q*.^[^ ^55^, ^57^ ^]^

A fully solution‐processed passive DBR‐DBR microcavity, targeted to feature an optical mode at *λ*_Opt._ ≈ 632 nm (*E*_Opt._ = 1.962 eV; see calculated transmission spectra shown in Figure 6B, dashed line), illustrates the importance of the above relations.^[^ ^32^ ^]^ For the design, Equation 2 was applied, using values measured for PFP and titanium oxide hydrates/PVAl hybrid films with 60 vol% inorganic content (annealed at 140 °C) as inputs for the refractive index of the low‐ and high‐refractive‐index DBR layers, as well as for the spacer layer separating the DBRs. The spacer layer's thickness can, then, be calculated so that the resonance condition *n*_cavity_(*λ*_Opt._)⋅*d*_cavity_ = *λ*_Opt._ (i.e., m = 2, see Equation 2) is satisfied. Reassuringly, a high transmission was experimentally recorded at the targeted optical mode wavelength (energy) for such a structure grown fully from solution (Figure 6B, solid line). The quality factor *Q* of the microcavity was found to be ≈50 (determined in transmission), which compares well with the value predicted by TMM of ≈70.^[^ ^32^ ^]^ A similar good agreement between calculation and experiment was demonstrated by Palo et al.,^[^ ^75^ ^]^ who used a titanium oxide hydrates/PVAl hybrid of ≈80 vol% inorganic content as the high‐refractive‐index material and Nafion^TM^—another commercially‐available fluorinated polymer—as the low‐refractive‐index material, to produce a microcavity with two 6‐bilayer DBRs. This structure led to a *Q* >90, as measured in reflectance. These *Q* factors are some of the highest reported values for solution‐processed planar microcavities comprising DBRs with less than 10 bilayers,^[^ ^67^, ^68^, ^69^, ^70^, ^71^, ^72^, ^73^, ^74^ ^]^ though we appreciate that a direct comparison is difficult to make. The reason is that the *Q* factors that can be realized in microcavities comprising DBRs as mirrors are highly dependent on the refractive index contrast between the high‐ and low‐refractive‐index layers in the DBRs and their number. Nonetheless, demonstration of such well‐functioning microcavities illustrates the many benefits that the titanium oxide hydrates/PVAl hybrids provide for the production of high‐quality photonic structures, including high and tunable refractive index, excellent film formation, reliable thickness control, as well as low optical loss. Furthermore, these demonstrations of fully solution‐processed microcavities suggest that structures with comparable *Q* factors as those reported for benchmark inorganic DBR‐DBR microcavities (*Q* ≈ 100–3000) may eventually be produced, if DBRs with a higher number of bilayers are utilized for their fabrication.^[^ ^32^ ^]^

As exciting, the relatively benign conditions needed to deposit titanium oxide hydrates/PVAl hybrid‐based DBRs open the possibility to study light‐matter interactions, e.g., mixed light‐matter states such as exciton‐polaritons, in material systems that cannot be readily incorporated into inorganic multilayer stacks because of their incompatibility with the DBR‐layers' deposition conditions. Strong light‐matter coupling between optical modes of fully solution‐processed DBR‐DBR microcavities and excitons from organic emissive layers, such as a perylene diimide derivative (*b*‐PDI‐1; Figure 6A, middle panel)^[^ ^32^ ^]^ and Rhodamine 6G,^[^ ^76^ ^]^ has already been reported. For example, in an active structure incorporating a *b*‐PDI‐1 emissive layer between two 4‐bilayer DBRs, an optical mode, *λ*_Opt._ ≈ 587 nm, was achieved at normal incidence, i.e., *E*_Opt._ $(\vec{k_{∥}}=\vec{0})=2.112\;\mathrm{eV}$, with a degree of detuning δ = *E*_Opt._ ∼ *E*_Exc._ = −59 meV 2.112 eV, with a degree of detuning δ = *E*_Opt._ − *E*_Exc._ = −59 meV with respect to the absorption maximum at normal incidence. Two polariton branches (diffuse blue halos in Figure 6C, top panel) were revealed by the energy dispersion (energy vs. in‐plane wavevector or output angle), consistent with the polariton eigenstates derived from a Jaynes‐Cummings Hamiltonian with a Rabi splitting, Ω=2·g=65meV (solid black lines, Figure 6C). Here, g denotes the light‐matter coupling constant. It is noteworthy that the energy dispersion simulated via the transfer matrix model accurately predicts the energetic positions and linewidths of the polariton branches (Figure 6C, bottom panel), reinforcing the notion that molecular hybrids, such as the titanium oxide hydrates/PVAl hybrid, facilitate the fabrication of fully solution‐processed microcavities of exceptional quality.^[^ ^32^ ^]^

## 4 Conclusion

The applications of inorganic/organic nanocomposites and hybrid materials are extensive because they can be immensely versatile with respect to composition, processing, and properties. Nanocomposites have been advanced with a variety of inorganic components, including inorganic clay compounds,^[^ ^77^, ^78^, ^79^, ^80^, ^81^ ^]^ metal oxo clusters,^[^ ^82^, ^83^, ^84^, ^85^ ^]^ oligosilsesquioxanes and their derivatives,^[^ ^86^, ^87^, ^88^, ^89^, ^90^ ^]^ mesoporous silica (zeolite),^[^ ^91^, ^92^, ^93^ ^]^ calcium carbonate,^[^ ^94^, ^95^ ^]^ and metallic^[^ ^96^, ^97^, ^98^ ^]^ or inorganic^[^ ^99^, ^100^ ^]^ nanoparticles, to name just a few. Despite significant global research efforts, achieving a good dispersion of the inorganic component within the organic matrix remains a challenge, especially at high loads, with nanoparticles in resins being one of the only nanocomposite systems leading to homogenous dispersions and permitting higher inorganic component loads.^[^ ^101^, ^102^ ^]^ As reviewed here, it seems beneficial to expand the materials library compatible with photonic crystal fabrication with molecular hybrids. Highly promising candidates include inorganic/organic hybrids based on titanium oxide hydrates and commodity polymers such as PVAl. Numerous 1D photonic structures have already been produced using these titanium oxide hydrates/PVAl hybrids as the high‐refractive‐index material alongside commodity plastics like PMMA, cellulose acetate (CA), Nafion™ or PFP as the low‐refractive‐index material. The properties of such distributed Bragg reflectors are summarized in **Table** **2**.

**Table 2:** Fully solution‐processed DBRs fabricated with the titanium oxide hydrates/PVAl hybrid (HyTiPVAl) as the high‐refractive‐index material.

Material system *n*_high_ / *n*_low_	Stopband center *λ*_c_ [nm]	Refractive index contrast Δ*n* at *λ*_c_ [–]	Number of bilayers [–]	Maximum reflectance [%]	Refs.
81 vol% HyTiPVAl/CA	750	0.40	10.5	85	[103]
79 vol% HyTiPVAl/Nafion^TM^	510	0.40	6	90	[75]
60 vol% HyTiPVAl/PMMA (annealed at 150 °C)	590, 741	0.34, 0.31	8.5	88, 82	[37]
60 vol% HyTiPVAl/PFP	730	0.36	10.5	98	[18]
60 vol% HyTiPVAl/PFP (annealed 130 °C)	600	0.50	10.5	>99	[18]
60 vol% HyTiPVAl/PFP (annealed 140 °C)	620	0.50	11.5	>99	[32]
80 vol% HyTiPVA/PMMA	528	0.26	7.5	94	
80 vol% HyTiPVA/PMMA	1225	0.20	7.5	76	
80 vol% HyTiPVA/PMMA (annealed 100 °C)	1078	0.29	7.5	91	

The excellent quality of such DBRs, which is comparable to inorganic structures produced by electron‐beam evaporation,^[^ ^32^ ^]^ positions them at the forefront for easy‐to‐coat, large‐area applications like heat mirrors and energy‐harvesting photonic structures for solar cells.^[^ ^37^ ^]^ Additionally, these DBRs are suitable for all‐optical temperature sensing because their stopband undergoes a wavelength shift and a measurable change in its reflectance that depend on the annealing temperature and that can be modeled precisely. Furthermore, DBR‐DBR optical microcavities can be produced layer by layer, fully from solution.^[^ ^32^, ^75^, ^76^ ^]^ These planar microcavities offer some of the highest *Q* factors for solution‐processed structures comprising DBRs with fewer than 10 bilayers ^[^ ^67^, ^68^, ^69^, ^70^, ^71^, ^72^, ^73^, ^74^ ^]^ and can exhibit exciton‐polaritons with a similar Rabi splitting as found for metal‐clad microcavities in which the same emissive layer was incorporated. The work by H. A. Qureshi et al., for instance, reported a Rabi splitting in solution‐processed structures that either matched or exceeded that of silver‐clad microcavities with Rhodamine 6G dispersed in PVAl as the emissive layer, reaching values as high as 400 meV.^[^ ^76^ ^]^ These initial activities promise to open a broad range of future opportunities for the control of light‐matter interactions. This includes options that harness polariton properties (e.g., a low effective mass, many‐body interactions, a delocalized nature, and their capability to display macroscopic coherence^[^ ^104^ ^]^), as well as the additional energetic pathways that polaritons provide, to modify light emission,^[^ ^105^, ^106^ ^]^ energy transfer,^[^ ^107^, ^108^ ^]^ and other photophysical processes in matter.^[^ ^109^ ^]^ Solution‐processed structures for lasing might be in reach as well.^[^ ^70^, ^110^, ^111^, ^112^, ^113^, ^114^ ^]^ Moreover, the ease with which the titanium oxide hydrates/PVAl hybrids can be patterned promises straightforward fabrication of 2D‐ or even 3D‐photonic architectures,^[^ ^31^, ^45^ ^]^ which are of interest, for example, for the production of optical waveguides, where light of specific wavelengths within the optical band gap of such structures can propagate loss‐less, also around tight corners and “kinks”,^[^ ^115^, ^116^ ^]^ and microcavity arrays, which are at the center of emergent quantum information technologies.^[^ ^117^ ^]^ Unambiguously, there is significant potential for further material design. Other metal oxide hydrates may be used for hybrid synthesis, such as zirconium or hafnium oxide hydrates, as well as mixed metal oxide hydrates derived from them. By exploiting the range of metal oxide hydrate precursors available, commonly used coating techniques like inkjet printing,^[^ ^118^, ^119^ ^]^ melt processing,^[^ ^120^, ^121^ ^]^ and additive manufacturing may become available for molecular hybrid deposition and photonic structure fabrication based on this class of materials. Some of these methodologies could open the development of thick optical structures based on metal oxide hydrates/poly(vinyl alcohol) hybrids for light in‐ and out‐coupling, packaging, and cladding, among other applications. Clear is that there remains ample unexplored territory with many possibilities yet to be discovered, especially when combining organic and inorganic systems with inorganic/organic molecular hybrids based on the large family of metal oxide hydrates to advance the forefront of optics and photonics.

## Conflict of Interest

The authors declare no conflict of interest.
